# Production of recombinant human procollagen type I C-terminal propeptide and establishment of a sandwich ELISA for quantification

**DOI:** 10.1038/s41598-017-16290-9

**Published:** 2017-11-21

**Authors:** Woo-Young Seo, Jeong-Ho Kim, Du-San Baek, Su-Jung Kim, Sujin Kang, Won Suk Yang, Ji-Ae Song, Moo-Seung Lee, Sunghoon Kim, Yong-Sung Kim

**Affiliations:** 1 Medicinal Bioconvergence Research Center, Seoul National University, Suwon, 16229 Korea; 2 Department of Molecular Science and Technology, Ajou University, Suwon, 16499 Korea; 3 Infectious Disease Research Center, Korea Research Institute of Bioscience and Biotechnology, Daejeon, 34141 Korea

## Abstract

Procollagen type I carboxy-terminal propeptide (PICP), derived from type I procollagen, has been identified as an indicator of type I collagen synthesis in bone matrix formation and skin recovery. PICP is a heterotrimeric glycoprotein consisting of two α1 chains (PICPα1) and one α2 chain (PICPα2). Here, we report the recombinant expression of human PICP using a mammalian expression system. Co-expression of PICPα1 and PICPα2 in HEK293F cells resulted in the production of functional PICP in the correctly assembled heterotrimeric form. Using the recombinant PICP as an antigen, we isolated PICP-specific human monoclonal antibodies from phage-displayed antibody libraries and raised rabbit polyclonal antibodies. Using those antibodies, we then developed a sandwich ELISA for PICP with a limit of detection of 1 ng/mL and a measurable range of 1–640 ng/mL. Both intra- and inter-assay imprecision values were <10%. For measuring PICP levels in human fibroblast cellular extracts and culture supernatants and a human serum, the developed ELISA kit displayed comparable performance to that of a commercialized kit. Our results provide an efficient production strategy for recombinant PICP, facilitating the generation of PICP-specific antibodies and development of PICP sandwich ELISA, with potential use in clinical diagnosis of serum samples and testing of cosmeceutical ingredients in fibroblast cell cultures.

## Introduction

Collagens are the major structural proteins of the extracellular matrix that play a crucial role in providing connective tissue structural integrity and homeostasis^1^. Particularly, they are highly associated with bone metabolism, wound healing, and skin recovery, and thus their synthesis is tightly controlled and regulated^2^. There are approximately 28 different types of collagens, all of which are composed of three polypeptide chains (α chains) forming either homotrimers with three identical α chains or heterotrimers with two/three different α chains^1^. Type I collagen is the most abundant human body collagen accounting for almost 90% of the organic portion of the bone matrix^2,3^. Therefore, analysis of type I collagen synthesis in the bone is critical to monitor both bone resorption and formation rates^2,4,5^.

**Figure 1:**
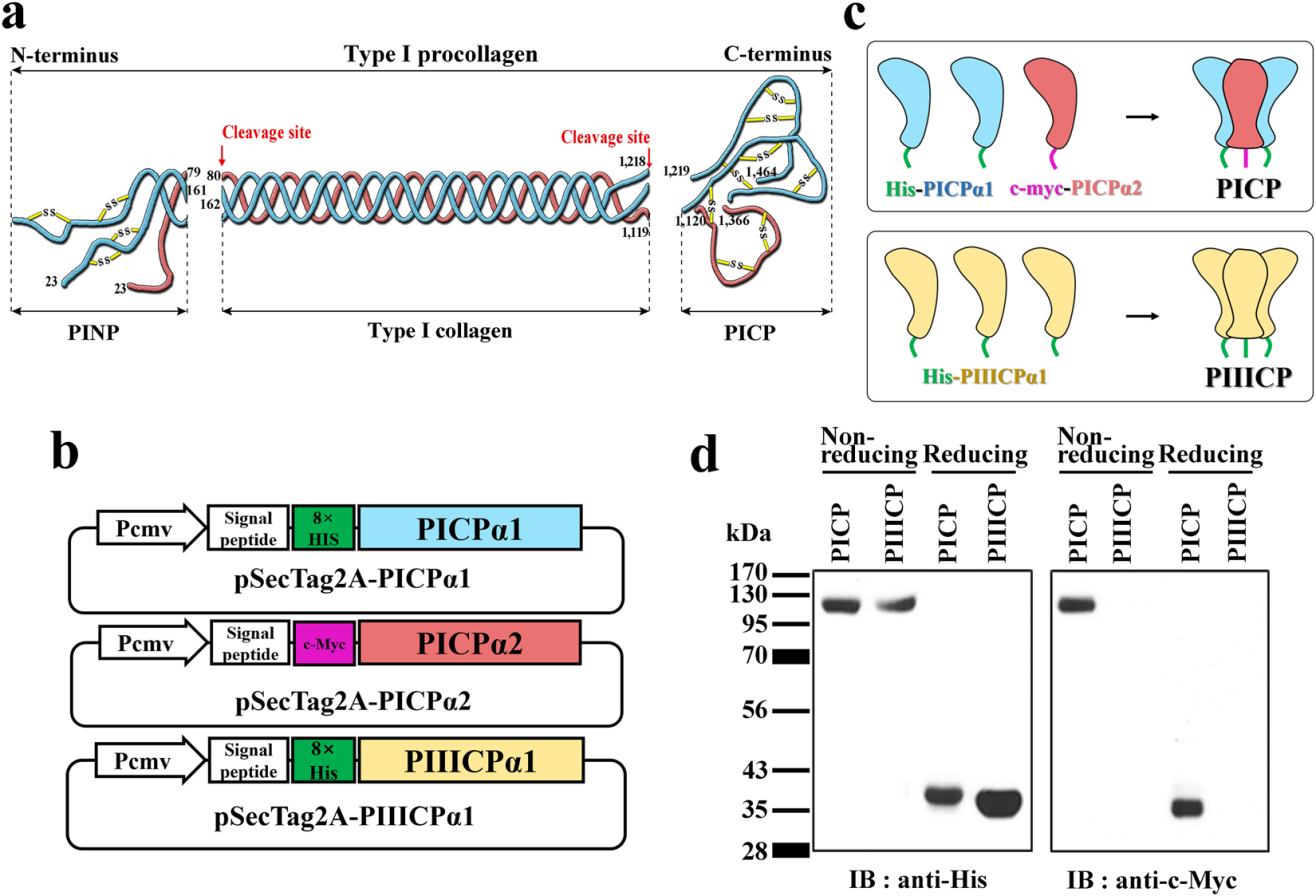
Design and expression of recombinant human PICP protein. (**a**) Schematic diagram showing the generation of PICP from type I procollagen by enzymatic cleavage (the red arrow indicates the cleavage site). PICPα1 and PICPα2 chains are represented as strings in cyan and magenta colors, respectively. Disulfide bonds are indicated with yellow lines. Each end of the strings is labeled with the residue number of type I procollagen. (**b**) Design of mammalian expression plasmids to secrete PICPα1, PICPα2, and PIIICPα1. The 8× His tag (green) and c-myc tag (purple) are indicated with colored rectangles. (**c**) Schematic diagram showing recombinant expression of the correctly assembled PICP and PIIICP. Two PICPα1 chains (cyan) and one PICPα2 chain (magenta) assemble into heterotrimeric PICP (upper), while three PIIICPα1 chains (light yellow) assemble into homotrimeric PIIICP (lower). (**d**) Western blot analysis of purified PICP and PIIICP. The proteins were subjected to SDS-PAGE in non-reducing or reducing conditions, and the gel was blotted with anti-His antibody (left) and anti-c-myc antibody (right). The corresponding full-length blots are shown in Supplementary Fig. 4.

The fibrillar type I and III collagens are derived from the precursor molecules, procollagens (~450 kDa), which are rod-like, triple-helical molecules (~300 nm in length) with globular amino (N)- and carboxy (C)-terminal propeptide domains^1,6^. While type III procollagen is a homotrimer comprising three identical α1 chains, type I procollagen is a heterotrimer consisting of two α1 chains and one α2 chain^1^. During its cellular expression and secretion, the procollagens are assembled in the trimeric form and then cleaved at specific N- and C-terminal sites by specific endopeptidases, generating three fragments: the procollagen type I N-terminal propeptide (PINP), type I collagen, and procollagen type I carboxy-terminal propeptide (PICP)^1,4^ (Fig. 1a). While the matured type I collagen polymerizes into extracellular collagen fibrils, PINP and PICP are released into the blood^4^. Unlikely with unstable PINP in the serum, PICP serum levels are stoichiometrically related to the amount of type I collagen synthesized in the bone matrix^3,7^. Accordingly, as a surrogate marker of type I collagen formation in the bone, the serum levels of PICP have been used as an indicator of bone formation and metabolism in physiological and disease conditions^3,8,9^. Furthermore, in the cosmetic industry, quantitative detection of PICP in fibroblast cell extracts and culture supernatants has been a routine procedure to assess the effects of cosmetic elements on the synthesis of type I collagen in the skin. Therefore, it is important to use a precise tool to quantify PICP in diverse samples, including the serum and fibroblast cell extracts.

PICP has been quantified using immunoassays since 1974^10,11^. For immunoassays, anti-PICP antibodies have been obtained by immunizing rabbits or mice with PICP purified from the culture media of human skin fibroblasts^10–13^. However, the isolation of PICP requires large amounts of cells, and additional steps for generating PICP from type I procollagen, such as treatment with bacterial collagenases^10,12,13^. Furthermore, at least two chromatographic steps are required for high purity and homogeneity to exclude structurally and biochemically similar molecules, such as procollagen type III C-terminal propeptide (PIIICP)^13^. Since these procedures are expensive, time-consuming, and laborious, recombinant expression of PICP is an alternative strategy for antibody isolation. However, the recombinant expression of PICP is not straightforward because of the challengeable structural feature of the heterotrimer consisting of two α1 polypeptide (PICPα1) chains and one α2 polypeptide (PICPα2) chain.

Here, we describe the recombinant expression and purification of human PICP using a mammalian cell expression system. Using recombinant PICP as an antigen, we then generated PICP-specific human monoclonal antibodies (mAbs) from a phage-displayed antibody library, and anti-PICP polyclonal antibodies (pAbs) by rabbit immunization. Using a combination of mAb and pAb, we finally developed a sandwich ELISA for the quantification of native PICP in various samples, including human fibroblast cell extracts and culture supernatants as well as human serum.

## Results

### Design of recombinant human PICP

PICP is a heterotrimeric glycoprotein with two PICPα1 chains and one PICPα2 chain, each of which has a single N-glycosylation site^7^ (Fig. 1a). Furthermore, each PICPα1 and PICPα2 chain has 8 cysteine residues at the conserved position of the collagen α chain^14^. Thus, PICP seems to be stabilized by intra- and inter-chain disulfide bonds in addition to non-covalent interactions at the chain interface (Fig. 1a). Considering these structural features, we chose a mammalian cell expression system for the recombinant expression of human PICP. We designed two mammalian secretion plasmids, encoding PICPα1 (residues 1218–1464) and PICPα2 (residues 1120–1366), respectively (Fig. 1b). To facilitate purification and validation of the heterotrimerization of the recombinant PICP, 8× His and c-myc tags were fused to the N-terminus of the PICPα1 and PICPα2 chains, respectively (Fig. 1b,c). For the expression of human homotrimeric PIIICP, we also constructed a mammalian expression plasmid encoding PIIICPα1 (residues 1222–1466) with an N-terminal 8× His tag (Fig. 1b,c).

### Expression and evaluation of recombinant PICP

Two plasmids, one encoding PICPα1 and the other encoding PICPα2, were transiently co-transfected at a 2:1 molar ratio into HEK293F cells^15^. After 7 days of culture, the secreted proteins were purified from the culture supernatants using Ni-NTA agarose column. The final yield of recombinant PICP, with purity more than 95%, was approximately 14 ± 3 mg/L, which is much higher than that (0.5–3 mg/L) obtained from human fibroblast cultures^10,13^. Recombinant PIIICP was also prepared by transient transfection into HEK293F cells, yielding 15 ± 1 mg/L of transfected cells. The purified proteins were analyzed by size-exclusion chromatography (SEC) to estimate the size and heterogeneity in solution under native conditions. Both PICP and PIIICP eluted mainly as a single peak on SEC with an apparent molecular weight roughly corresponding to the expected heterotrimer and homotrimer, respectively (Supplementary Fig. 1). Though PICP and PIIICP exhibited a shoulder and minor peaks that eluted earlier than the native trimer peak representing the oligomer species, respectively, they did not show eluted peaks corresponding to unassembled free dimers or monomers (Supplementary Fig. 1). Next, the purified proteins were analyzed by western blotting using anti-His or anti-c-myc antibodies (Fig. 1d). For purified PICP, both anti-His and anti-c-myc antibodies detected a single band at the expected heterotrimer size of PICP (~120 kDa) in non-reducing conditions. In reducing conditions, however, each antibody detected only one band corresponding to the 8× His-tagged PICPα1 monomer (~40 kDa) and c-myc-tagged PICPα2 monomer (~36 kDa), respectively. For purified PIIICP, only anti-His antibody, but not anti-c-myc antibody, detected a single band corresponding to the homotrimer (~120 kDa) and its 8× His-tagged PIIICPα1 monomer (~37 kDa) in non-reducing and reducing conditions, respectively (Fig. 1d). These results suggested that PICP and PIIICP were purified into their heterotrimeric and homotrimeric forms, respectively, in the mammalian cell expression system. One step further purification of the Ni-NTA agarose-purified PICP using an anti-c-myc antibody-conjugated agarose bead confirmed that PICP was dominantly expressed in the heterotrimeric form without noticeable amount of free monomers or homotrimers (Supplementary Fig. 2).

**Figure 2:**
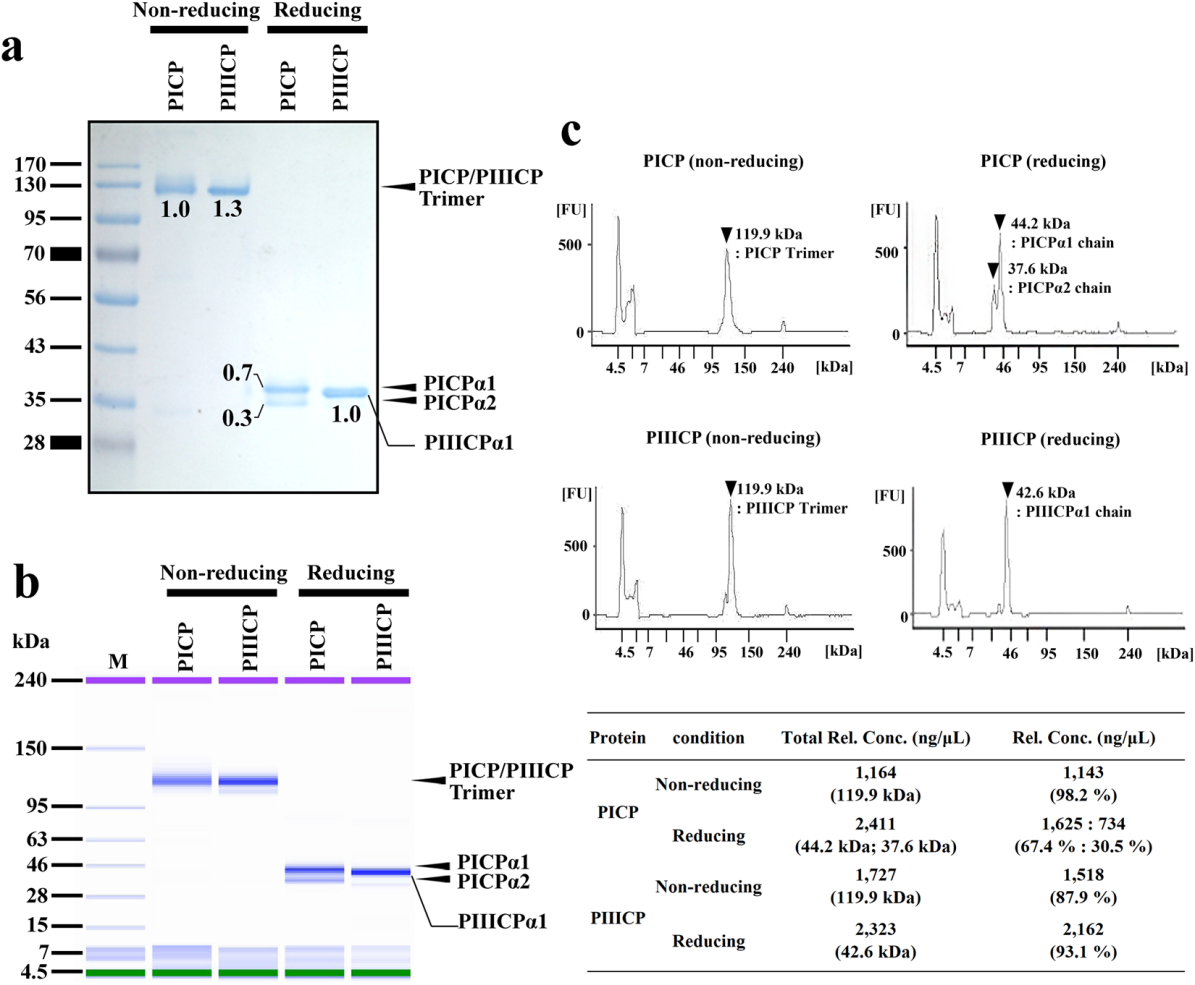
SDS-PAGE and SDS-CGE analyses of purified recombinant PICP and PIIICP. (**a**) Non-reducing and reducing SDS-PAGE analyses of the purified recombinant PICP and PIIICP. The arrows dictate the eluted position of indicated proteins. The number below the band indicates the relative value of band intensity of each band to that of PICP heterotrimer band in the non-reducing conditions. (**b**) Gel-like image of SDS-CGE analysis of the purified recombinant PICP and PIIICP using a Protein 230 assay kit. In non-reducing conditions, PICP and PIIICP appeared mainly as a single band at the expected size. In reducing conditions, PICP and PIIICP showed two bands for PICPα1 and PICPα2 and a single band for PIIICPα1, respectively. (**c**) Electropherogram of SDS-CGE analysis of the recombinant PICP and PIIICP shown in (**b)**. Significant peaks are marked with black arrow heads, with the calculated molecular weights. The inset table below shows the precise quantity and ratio of each peak area in the electropherogram. The raw images of SDS-CGS analysis are shown in Supplementary Fig. 5.

To determine the molecular composition ratio, the purified heterotrimeric PICP was analyzed by sodium dodecyl sulfate-polyacrylamide gel electrophoresis (SDS-PAGE) and SDS-capillary gel electrophoresis (SDS-CGE). The purified recombinant PICP or PIIICP was eluted, showing a single band of size ~120 kDa in non-reducing SDS-PAGE (Fig. 2a) and SDS-CGE analyses (Fig. 2b,c and Supplementary Fig. 3), corresponding to the trimeric molecular weight. The apparent molecular weight was consistent with that of PICP purified from human fibroblast cultures^10,13^. Under the reducing conditions, homotrimeric PIIICP showed only one band with an apparent molecular weight roughly corresponding to the 8× His-tagged PIIICPα1 monomer (Fig. 2a,b). The two analyses of PICP under reducing conditions resulted in two distinct bands with apparent molecular weights roughly corresponding to the 8× His-tagged PICPα1 monomer and c-myc-tagged PICPα2 monomer, respectively (Fig. 2a,b). Next, by quantifying the relative band intensity on SDS-PAGE gel and the peak area of SDS-CGE electropherogram of the proteins, which correspond to the amount of protein in the band, we analyzed the ratio of the two separated bands observed in the PICP lane under reducing conditions. Intriguingly, both band intensity and peak area of the two bands represented a ratio of approximately 7:3 (Fig. 2a,c and Supplementary Fig. 3), which is almost the same composition ratio of natural heterotrimeric PICP (PICPα1 chain: PICPα2 chain = 2:1 molar ratio)^10,13^. These results suggest that recombinant PICP was dominantly expressed in the correctly assembled heterotrimeric molecule composed of two PICPα1 chains and one PICPα2 chain, like in naturally occurring PICP^10,13^. Nonetheless, since the molar ratio of PICPα1 chain to PICPα2 chain was slightly higher (approximately 17%) and the possible homotrimer of PICPα1 is indistinguishable from the heterotrimer of PICP in the above analytical methods, the presence of PICPα1 homotrimer in the purified PICP cannot be completely excluded.

### Screening and selection of PICP-specific antibody using recombinant PICP

**Figure 3:**
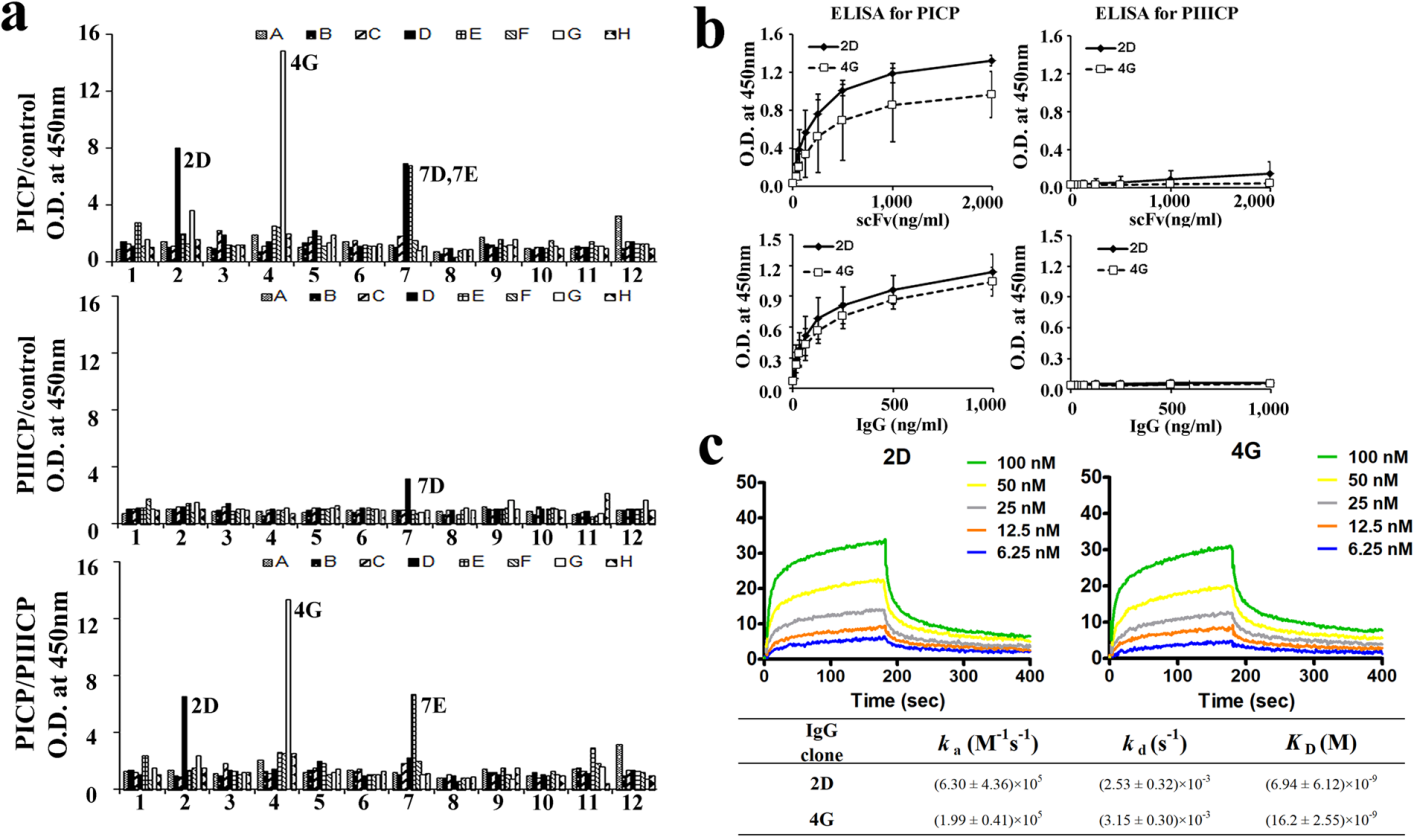
Isolation and characterization of anti-PICP 2D and 4G mAbs. (**a**) Phage ELISA of isolated individual phage to determine the binding activity of plate-coated PICP (top), PIIICP (middle), and PICP with its soluble competitor PIIICP (bottom). Phages were isolated after 4 rounds of panning of human scFv phage library against recombinant PICP in the presence of competitor PIIICP. (**b**) Indirect ELISA of 2D and 4 G clones in the format of scFv (0~2 μg/mL) and IgG1 (0~1 μg/mL) to determine their binding specificity to plate-coated recombinant PICP (left) and recombinant PIIICP (right). (**c**) Representative SPR sensorgrams showing the kinetic interactions of IgG1-formatted 2D and 4 G mAbs with recombinant PICP. The inset table shows the kinetic interaction parameters. Each value represents the mean ± SD of five data sets.

To generate antibodies for a PICP-quantification sandwich ELISA system, we first isolated anti-PICP mAbs by screening a large (>10^9^ diversity), fully synthetic human single chain variable fragment (scFv) phage library^16^ against the purified recombinant PICP. We performed 4 rounds of phage panning against recombinant PICP. During the panning, recombinant PIIICP, a homologous protein with ~60% sequence identity with PICP^6,14^, was used as a soluble competitor to isolate PICP-specific antibodies. Three positive clones (2D, 4 G, and 7E) with high specificity to PICP were identified by phage ELISA (Fig. 3a). The higher binding clones of 2D and 4 G were purified from bacterial supernatants using Ni-NTA resin, and then tested by indirect ELISA against recombinant PICP. Results showed specific and dose-dependent binding to recombinant PICP, but not to recombinant PIIICP (Fig. 3b top). Based on these results, 2D and 4 G scFv clones were converted into human IgG1 format by subcloning the VH and VL genes into plasmids carrying the constant genes of heavy and light chains^17^, respectively. Two 2D and 4 G IgG1 mAbs were then expressed in HEK293F cells and purified from the culture supernatants using a protein A resin^15^. The purified mAbs also showed specific interaction with PICP (Fig. 3b bottom). Quantitative analysis of the binding parameters by surface plasmon resonance (SPR) showed that 2D and 4 G mAbs specifically bound to PICP with equilibrium dissociation binding constants (*K*_D_) of ~6.9 nM and 16.2 nM, respectively (Fig. 3c). Since 2D showed higher affinity than 4 G, we decided to use 2D mAb for further characterization.

To obtain an anti-PICP pAb, we immunized rabbits with the recombinant human PICP. We obtained anti-PICP rabbit pAb by purification from the serum using a protein A resin and then a PICP affinity column.

### Isolated 2D mAb specifically binds to native PICP

**Figure 4:**
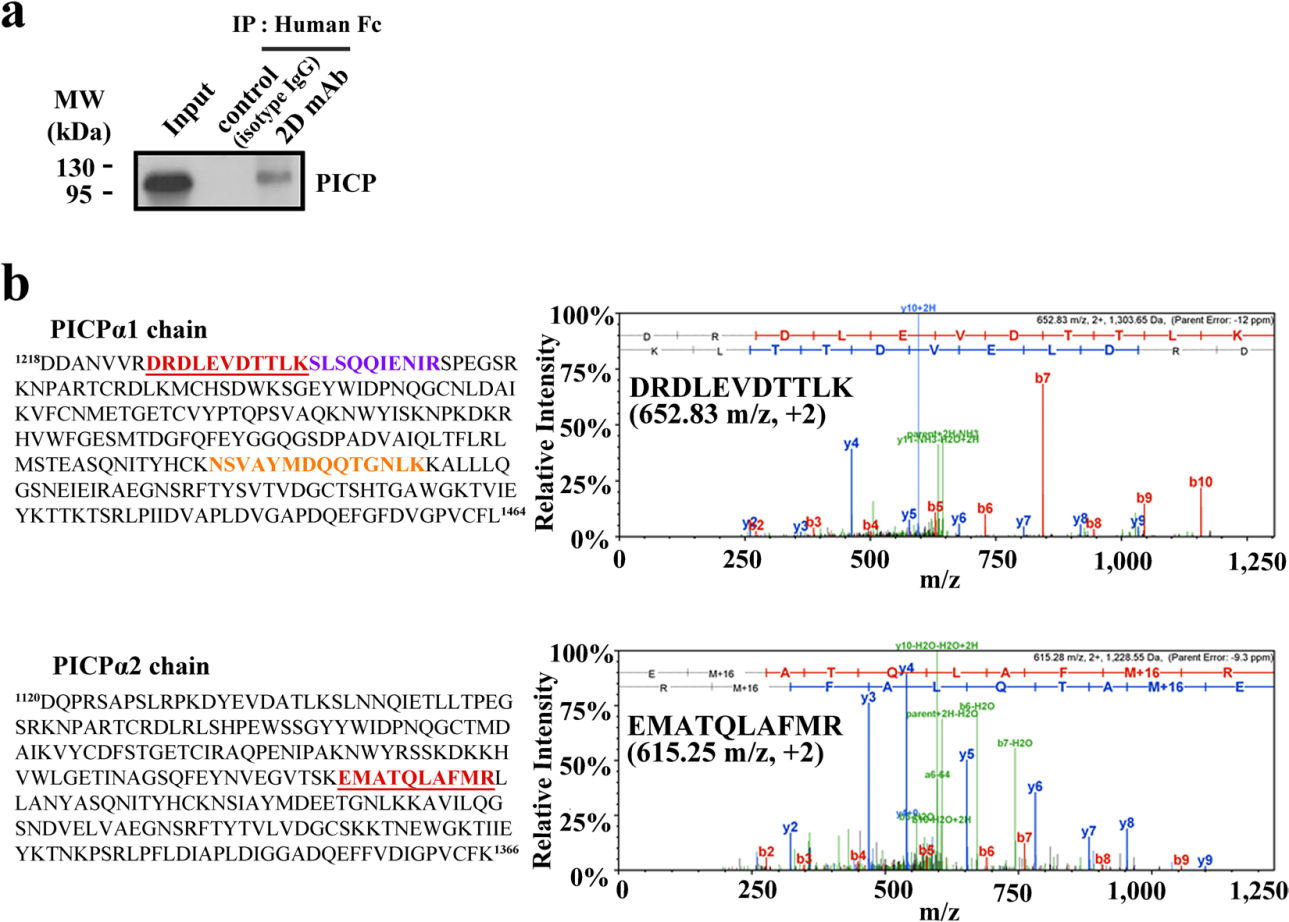
Specific binding of 2D mAb to native PICP. (**a**) Western blot analysis of the anti-PICP 2D mAb immunoprecipitation (IP) for culture supernatants of primary dermal fibroblast cells. The corresponding full-length blots are shown in Supplementary Fig. 6. (**b**) Identification of PICP protein in the immunoprecipitated gel band by LC/MS. The three different peptides and one peptide identified from the PICPα1 and PICPα2 chains, respectively, were indicated in distinct colors in the amino acid sequence. The left panels show the representative MS/MS spectrum for the identified peptides of DRDLEVDTTLK (652.83 m/z, +2) from PICPα1 (top) and EMATQLAFMR (615.28 m/z, +2) from PICPα2 (bottom), underlined in each sequence.

To determine whether 2D mAb binds to native PICP, we performed immunoprecipitation using 2D mAb for the culture supernatants of human primary dermal fibroblast cells. 2D mAb, but not an isotype control IgG antibody, precipitated native PICP from the primary fibroblast cell supernatant (Fig. 4a), demonstrating the specificity of 2D mAb to natural PICP.

To confirm whether the protein immunoprecipitated by 2D mAb was the native heterotrimeric PICP, we excised the gel band detected in western blotting (Fig. 4a), performed an in-gel digest with trypsin, and analyzed the peptides by liquid chromatography–mass spectrometry (LC/MS). Thus, 3 unique peptides from PICPα1 and 1 unique peptide from PICPα2 were identified by LC/MS analysis, respectively (Fig. 4b). Figure 4b also shows the representative MS/MS spectrum for the identified peptides that originated from the PICPα1 (^1226^DRDLEVDTTLK^1236^, 652.83 m/z) and PICPα2 (^1249^EMATQLAFMR^1258^, 615.28 m/z) chains. These results strongly suggest that the isolated 2D mAb is specific to natural PICP, and hence the recombinant PICP can be used as a high-quality antigen to screen anti-PICP antibodies as a substitute to PICP purified from human fibroblast cell cultures.

### Establishment of a sandwich ELISA for PICP quantification

**Figure 5:**
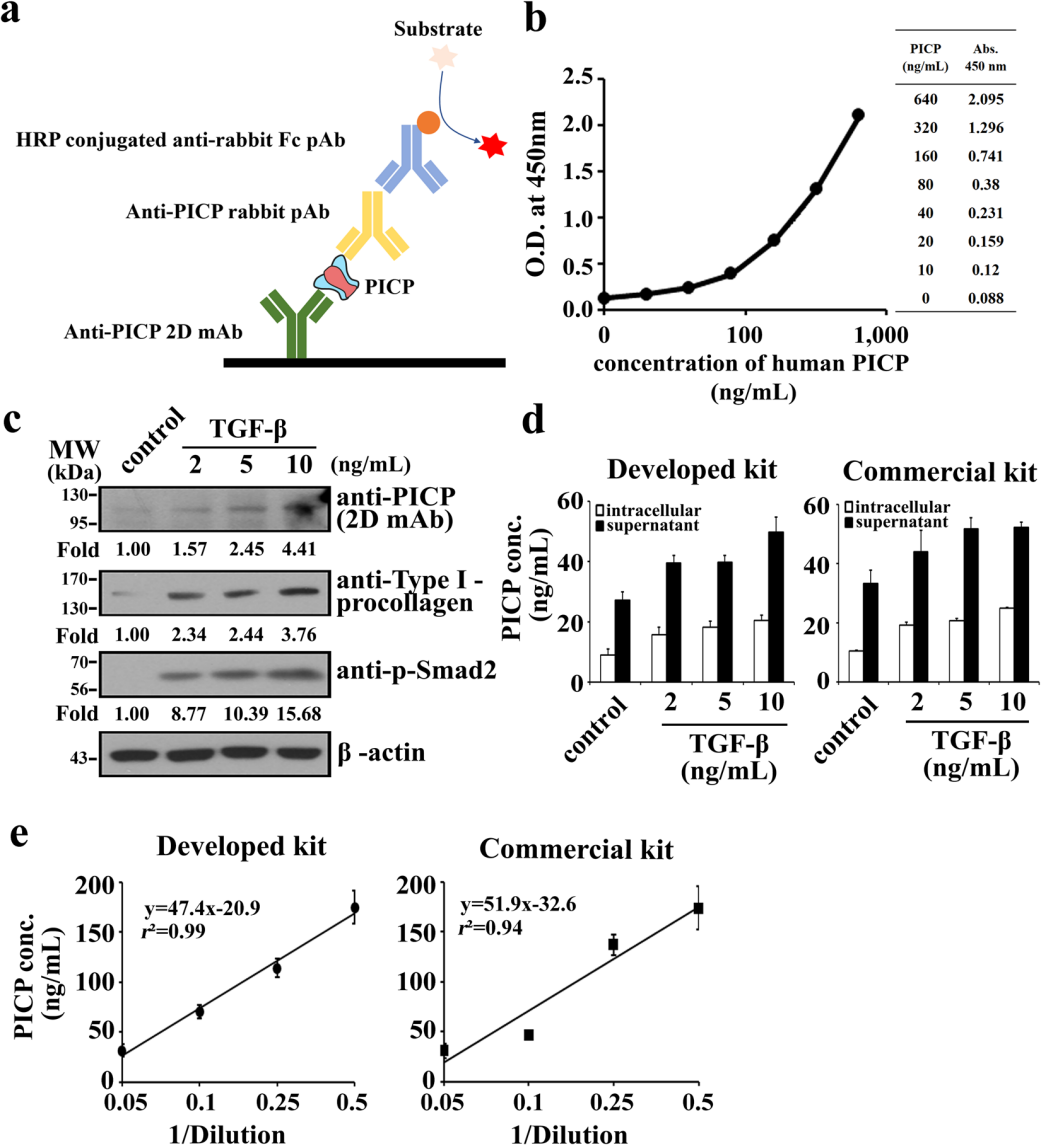
Establishment and validation of a sandwich ELISA for PICP quantification. (**a**) Schematic representation of the developed sandwich ELISA (see text for details). (**b**) Standard curve of the developed sandwich ELISA for quantification of recombinant human PICP. The data was fitted into a 4PL model to assess the correlationship between absorbance and the increasing concentration of recombinant PICP (*r* ^2^ = 0.99). (**c**) Western blotting to monitor the levels of PICP, type 1 procollagen, and phosphorylated Smad2 in LX-2 cells. For western blot analysis, an equal quantity of LX-2 cellular lysate was loaded using β-actin as a loading control. The number below the panel indicates the relative value of band intensity of the proteins compared with that of control after normalization of the band intensity to that of β-actin for each sample. The corresponding full-length blots are shown in Supplementary Fig. 7. (**d**) Quantification of PICP levels in the cellular extracts and culture supernatants of TGF-β stimulated LX-2 cells using the developed sandwich ELISA and the commercially available Takara ELISA kit (MK101). Error bars, ±SD (n = 3). (**c**,**d**) LX-2 cells were treated with medium (control) or the indicated concentrations of TGF-β for 12 h, and then the soluble cellular extracts and culture supernatants were subjected to the analyses. (**e**) Measurements of PICP levels in a normal human serum diluted at the indicated rate using the developed sandwich ELISA kit and the commercially available Takara ELISA kit (MK101). Error bars, ±SD (n = 3). Inset shows the equation for the fitted line and the correlation coefficient value (*r* ^2^).

Using a combination of anti-PICP human 2D mAb and rabbit pAb, we sought to develop a PICP sandwich ELISA. For sandwich ELISA, many reports have suggested that a combination of mAb as the capture antibody and pAb as the detection antibody is most effective for a highly sensitive and specific assay^18,19^. The reason is that mAb can specifically capture the target antigen with high affinity from a mixture of diverse proteins, such as serum or cell extracts. After the washing steps, the captured antigen may have more opportunity to be detected by a pAb rather than another mAb, since pAb is a composite of antibodies with different epitopes while maintaining the antigen specificity^20^. In other words, pAbs can recognize diverse epitopes of antigen, which are exposed and accessible after the binding of a capture mAb. Therefore, we used 2D mAb as the capture antibody, and anti-PICP rabbit pAb as the detection antibody (Fig. 5a).

To develop a highly sensitive sandwich ELISA, we optimized the quantity of the capture 2D mAb to be coated on the plate (0.4 mg), and the dilution factor of the detecting pAb (1:10,000) out of 1 mg/mL. The color was developed using a goat horseradish peroxidase (HRP)-conjugated anti-rabbit IgG secondary antibody. The standard calibration curve showed a good correlationship between absorbance and the increasing concentration of recombinant PICP (*r* ^2^ = 0.99), when it was fitted into a four parameter logistic (4PL) model^19^ (Fig. 5b). The 4PL model is the most recommended fitting model for concentration-response calibration curves in immunoassays^19^. According to the standard curve, the measurable detection range of our developed sandwich ELISA was 1–640 ng/mL. The limit of detection (LoD), an analytical sensitivity parameter, was determined to be 1 ng/mL, as calculated by the widely used formula: LoD = average absorbance of the blank +3 × [SD (standard deviation) of the blank]^19,21^. When PIIICP, which is highly homologous with PICP^22^, was used as an antigen at the same concentration as that of PICP, it remained undetected, demonstrating the specificity of our ELISA system.

To assess the accuracy and reproducibility of the developed ELISA, recovery tests were performed with recombinant PICP at different concentrations (640, 320, 160, 80, 40, 20, and 10 ng/mL). The percentage of recovery rate was calculated by the following formula: [the measured value/expected value × 100]^18,21^, where the expected value was determined using a BCA Kit (Pierce, 23225). The recovery rate ranged from 80.3 to 95.1% (Table 1). When the coefficient of variation (CV), a precision parameter, was determined by the formula: [SD of the mean/the mean value × 100]^19,21^, it showed the acceptable range with less than 10% (Table 1). To assess the intra- and inter-assay variability of the developed ELISA, we measured the CV for the assays by measuring solutions containing three spiked doses of PICP in phosphate buffered saline (PBS)^18^. The CV values for both assays were less than 10% and considered acceptable (Table 2).

**Table 1:** The recovery rate of the developed sandwich ELISA kit.

Expected concentration (ng/mL)^a^	Measured concentration (ng/mL)^b^	CV (%)^c^	Recovery rate (%)^d^
640	549.3 ± 32.1	5.8 ± 0.3	85.9 ± 5.0
320	266.7 ± 18.9	7.1 ± 0.5	83.3 ± 5.9
160	140.3 ± 2.8	2.0 ± 0.1	87.7 ± 1.8
80	76.1 ± 3.4	4.5 ± 0.2	95.1 ± 4.2
40	32.2 ± 2.3	7.2 ± 0.5	80.5 ± 5.8
20	16.1 ± 0.8	5.0 ± 0.3	80.3 ± 4.0
10	9.1 ± 1.3	4.8 ± 0.2	91.1 ± 4.3

^a^The expected concentrations were determined using a BCA Kit (Pierce). The values are an average of 3 independent measurements in triplicates.

^b^The measured concentration using the developed ELISA kit in this study.

^c^CV (coefficient of variation) value was determined as percentage by the equation: [SD of the mean value/mean value × 100].

^d^The recovery rate was calculated as percentage by the formula: [measured value/expected value × 100].

^b,c,d^Data represent the mean ± SD (standard deviation) for at least 3 independent experiments performed in triplicates.

**Table 2:** Coefficient of variation (CV) for intra-assay and inter-assay^a^.

Samples	Intra-assay precision		Inter-assay precision	
	Measured concentration (ng/mL)	CV (%)	Measured concentration (ng/mL)	CV (%)
A	46.6 ± 3.0	6.4 ± 0.4	49.1 ± 2.7	5.6 ± 0.3
B	24.1 ± 2.3	9.5 ± 0.9	23.8 ± 1.8	7.6 ± 0.6
C	11.9 ± 1.0	8.5 ± 0.7	12.3 ± 1.0	7.9 ± 0.6

^a^Intra-assay precision was calculated from four independent experiments of the same assay on a single day but at different time points, while inter-assay precision was calculated from the assay repeated on three different days (n = 3). All the assays were carried out in triplicate. Data are expressed as mean ± SD (standard deviation). CV: Coefficient of variation.

### Evaluation of the developed sandwich ELISA

To validate our developed sandwich ELISA kit, it was tested to determine PICP concentrations in cellular extracts of human fibroblast LX-2 cells^23^, and compared to those of a commercialized PICP sandwich ELISA kit from Takara Inc. (Cat. #MK101). The cells were treated with transforming growth factor-beta (TGF-β), the key mediator of human fibrogenesis^23^, to stimulate collagen production^24^, and the soluble cellular extracts and culture supernatants were then subjected to western blotting and/or ELISA assays. Western blotting of the TGF-β-treated cellular extracts revealed increased production of type I procollagen, phosphorylated Smad2, and PICP in proportion with TGF-β concentration (Fig. 5c), confirming the dose-dependent response of LX-2 cells to TGF-β^23^. Both our developed and Takara’s sandwich ELISA kits revealed the TGF-β-mediated dose-dependent increase in PICP levels in the cellular extracts and culture supernatants (Fig. 5d), consistent with the data obtained from western blotting (Fig. 5c). The observation of higher PICP levels in the fibroblast culture supernatants than those in the cellular extracts was in good agreement with the previous result^25^.

We further validated our developed sandwich ELISA kit by measuring PICP levels in a normal human serum from healthy humans. Our developed and Takara’s sandwich ELISA kits exhibited linearly decreased PICP levels in proportion to the serum dilution rate with correlation coefficient values of *r* ^2^ ≈ 0.99 and *r* ^2^ ≈ 0.94, respectively (Fig. 5e). The measured PICP levels by the developed and Takara’s sandwich ELISA kits were 267 ± 80 ng/mL and 243 ± 59 ng/mL, respectively, which belongs to the ranges detected in normal human adult serum (about 50–350 ng/mL)^9,10,26^. Taken together, the above results demonstrate that the performance of the developed ELISA kit for the practical sample was comparable to that of the commercialized Takara kit.

## Discussion

To date, heterotrimeric PICP has been prepared from the culture supernatants and cellular extracts of human fibroblast cells, and used as an immunogen for the development of anti-PICP antibodies^10–13^. However, the conventional purification method is not efficient to produce large amounts of PICP with high purity, mainly because of the low collagen expression levels in the fibroblasts, an additional enzymatic cleavage step required to generate PICP from procollagen, and stepwise purification procedures to exclude the homologous proteins, such as PIIICP^10,12,13^. In this study, we demonstrated the functional expression of recombinant human PICP using the mammalian expression system (HEK293F). For recombinant expression, we designed two mammalian secretion plasmids encoding PICPα1 and PICPα2, respectively, and co-expressed them by co-transfection of the two plasmids in a 2:1 molar ratio into the cells. The 8× His tag fused to the PICPα1 chain facilitated one step purification of trimeric PICP with more than 95% purity, and a purification yield of approximately 14 mg out of 1-L culture. The c-myc tag fused to the N-terminus of the PICPα2 chain was exploited to monitor the assembly with His-tagged PICPα1 in the recombinant PICP by western blotting. SDS-PAGE and SDS-CGE analyses demonstrated almost the same composition of recombinant PICP with that of naturally occurring PICP^10,13^, consisting of two PICPα1 chains and one PICPα2 chain.

Intriguingly, the co-expression of two PICPα1 chains and one PICPα2 chains in mammalian cells resulted in the generation of heterotrimeric PICP with the correct molar composition. Intracellular assembly of fibrillar procollagen from its three polypeptide chains is initiated by association of the C-propeptide domains^1,27^. Despite the co-expression of several procollagen types in the same cells, each type of procollagen precisely assembles with the cognate polypeptide α chain^1^. This strict chain selectivity during the intracellular trimerization of different collagen types is conferred by the C-propeptide domains, particularly by a highly variable discontinuous sequence of 15 amino acids within the domains, called the chain recognition motif^14,28^. PICPα1 and PICPα2 have the 15-residue chain recognition motif of ^1339^GGQGSDPADVAI^1350^..^1359^STE^1361^ and ^1241^NVEGVTSKEMAT^1252^..^1261^ANY^1263^, respectively^29^. When expressed alone, type I procollagen α1 chain has the ability to form the homotrimer with low efficiency, while the α2 chain homotrimer has not been detected^1,30^. A recent study has also shown that recombinant expression of PICPα1 alone, but not PICPα2, results in the formation of homotrimer^31^. However, co-expression of PICPα1 and PICPα2 yield the well-folded PICP heterotrimer^31^. Thus, co-expression of PICPα1 and PICPα2 within the same cells seems to be required and sufficient for the correct heterotrimeric assembly of PICP, explaining our successful recombinant expression of human PICP.

While the crystal structure of recombinant homotrimeric PIIICP is available^6,14^, the three-dimensional structure of heterodimeric PICP has not yet been determined. Our study of recombinant expression of PICP may facilitate large-scale preparation for three-dimensional structure determination. Further, by exploiting the very stable heterodimeric structure of PICP^31,32^, recombinant PICP may be utilized as a stable heterotrimer scaffold for two distinct proteins to be assembled into a heterotrimer with a 2:1 molar ratio by N-terminal fusion to the PICPα1 and PICPα2 chains, respectively. The carboxy-terminal telopeptide of type I collagen (ICTP), which is produced when the collagen is degraded, has been monitored as a diagnostic biomarker in the serum for bone collagen degradation^33^. While disulfide-linked heterotrimeric PICP is released during biosynthesis of type I collagen, cross-linked ICTP is cleaved from matured collagen^33^. Heterotrimerization of ICTP by cross-linking occurs outside of cells by a series of post-translational modifications during assembly and cross-linking of matured collagen for the formation of collagen fibrils and collagen fibers^34^. Thus, recombinant co-expression of ICTPα1 (residues 1193–1217) and ICTPα2 (residues 1105–1119) fragments in HEK293F cells is not likely to produce correctly assembled heterotrimeric ICTP. However, co-expression of PICPα1 (residues 1218–1464) and PICPα2 (residues 1120–1366) with the N-terminal upstream regions to include the respective ICTP chain could generate the correctly assembled heterotrimeric ICTP-PICP protein due to the heterotrimerization of PICP. Then the recombinant heterotrimeric ICTP-PICP protein may be used as a screening antigen for isolation of ICTP-specific antibodies using PICP as a competitor.

High quality antigen preparation is essential for the development of specific antibodies. Our preparation of recombinant PICP also achieved high purity and homogeneity, making it suitable for use as an antigen in phage display as well as animal immunization to obtain antibodies. Furthermore, we obtained three highly PICP-specific mAbs. Clone 2D, having the highest affinity, could be used as the capture antibody in the PICP sandwich ELISA. The specificity of 2D mAb to natural PICP from fibroblast cells was confirmed by immunoprecipitation, followed by peptide analysis by LC/MS. We also immunized rabbits with our recombinant PICP and generated an anti-PICP rabbit pAb, which served as the detection antibody in the sandwich ELISA. Hence, our results suggest that the purified recombinant PICP can be used as a substitute for native PICP.

Synthesis of type I collagen generally occurs during bone metabolism and tissue regeneration, and its rate is highly correlated with metabolic bone diseases or to the extent of wound healing. Furthermore, the induction of type I collagen synthesis in human fibroblast cells is an important criteria for evaluating the function of cosmetic ingredients. Thus, an accurate quantification of PICP, as an alternative to type I collagen, is essential for clinical diagnosis of plasma and serum samples, and for testing the cosmeceutical ingredients in cultured cell extracts and supernatants. There are several commercially available sandwich ELISA kits for quantification of PICP having a different detection range and sensitivity. For examples, Takara (MK101) and LSBio (LS-F12337) kits have a detection range of 10–640 ng/mL and 31.2–2,000 pg/mL, respectively. We also established a sandwich ELISA kit for quantification of PICP using a combination of anti-PICP human 2D mAb as the capture antibody and rabbit pAb as the detection antibody. The measurable detection range of our developed sandwich ELISA kit is 1–640 ng/mL, which is similar to that of Takara kit, and it has a more sensitive LoD (1 ng/mL) compared to that of Takara kit (10 ng/mL). The high recovery rates (80–95%) and low CV values (<10%) for inter- and intra-assay suggest that the developed sandwich ELISA has an acceptable performance and can be used as a reliable tool for quantification of PICP. To quantify PICP levels in the cellular extracts and culture supernatants of fibroblast cells as well as in a human serum, the developed sandwich ELISA displayed comparable performance to that of the commercialized Takara kit. This indicates that the developed sandwich ELISA kit may be a useful tool for screening cosmeceutical ingredients in fibroblast cell cultures to evaluate their function. The PICP concentration in normal human adult serum is normally about 50–350 ng/mL^9,10,26^. Therefore, the sandwich ELISA for human PICP established in this study could also be applicable for clinical diagnosis of serum samples from patients with minimal dilution.

## Materials and Methods

### Construction of PICP and PIIICP expression vectors

The cDNA plasmids carrying the complete PICPα1 and PICPα2 chain genes were provided by KRIBB (Korea Research Institute of Bioscience and Biotechnology). DNA encoding the recombinant PICPα1 chain (residues 1218–1464) was amplified from the cDNA vector using the forward primer: 5′-AAA AGG CGC GCC CAT CAC CAT CAC CAT CAC CAT CAC GGC GCC GAT GAT GCC AAT GTG GTT CG-3′ and the reverse primer: 5′-TTG GGC CCT TAC TAC AGG AAG CAG ACA GGG C-3′. The forward primer encodes 8× His tag (underlined). DNA encoding the PICPα2 chain (residues 1120–1366) was obtained by overlapping polymerase chain reaction (PCR) to remove a restriction enzyme site by a silent mutation using the primers: 5′-AAA AGG CGC GCC GAA CAG AAA CTG ATC TCT GAA GAA GAC CTG GGC GCC GAC CAG CCT CGC TCA G-3′, 5′-GGA TGT TTT CAG GTT GGG CAC GGA TAC AGG TTT CGC C-3′, 5′-GCC CAA CCT GAA AAC ATC C-3′, and 5′-TTG GGC CCC TAT TAT TTG AAA C-3′. The first forward primer for PICPα2 chain encodes c-myc tag (underlined). All PCR reactions were performed with a 25 ng template DNA, 200 μM dNTP, 0.25 μM forward primer, 0.25 μM reverse primer, 1 × PCR buffer, and 1.5 unit of speed pfu polymerase (Nanohelix) in 50 μL total reaction volume. Amplified DNAs were then purified after electrophoresis on 1.2% agarose gel, and inserted into the pSecTag2A mammalian expression vector^15^, using *Asc*I/*Apa*I sites.

### Expression and purification of PICP and PIIICP

The constructed plasmids encoding the PICPα1 and PICPα2 chains were transiently co-transfected at a 2:1 molar ratio into HEK293F cells in FreeStyle 293 media (Invitrogen), ranged from 200-mL to 1000-mL culture volume, following a previously described standard protocol^15,17^. HEK293F cells grown overnight were centrifuged prior to transfection and resuspended at a cell density of 2 × 10^6^ cells/mL in Freestyle 293 media. The plasmid DNAs mixed at the 2:1 molar ratio were resuspended in the FreeStyle 293 media and sterilized by filtration (0.2 µm pore size, cellulose acetate; Corning, 431219). Stock solution (1 mg/mL) of a 40-kDa linear polyethyleneimine (PEI, Polysciences, 24765) was prepared in water, neutralized to be pH 7.4 with HCl, sterilized by filtration (Corning, 431219). The DNA/PEI cocktail was prepared in FreeStyle 293 media by mixing DNA and PEI to be final amount of 1.25 µg of DNA and 3.75 µg of PEI (quantities are per mL of the culture to be transfected) and gently mixed by 2 times inverting and incubated for 10 min at 25 °C. Then the DNA/PEI cocktail (in a volume equivalent to one-tenth of the culture to be transfected) was slowly added to the prepared cells. For the expression of PIIICP, the plasmid DNA encoding PIIICPα1/PEI cocktail was transfected into HEK293F cells in the same way. Cells were cultured in Erlenmeyer flasks using 20% of the nominal volume at 125 ± 10 r.p.m. under humidified conditions (37 °C and 8% CO_2_ atmosphere). After 7 days of culture, the culture supernatants were harvested by centrifugation and filtration (pore size: 0.22 µm, cellulose acetate; Corning CLS430521). Recombinant PICP and PIIICP were purified from the supernatants using 1 mL of Ni-NTA resin (GE Healthcare). Filtered supernatants were incubated with the resin for 1 h at 4 °C under continuous rotation (4 rpm). After washing the resin with washing buffer (PBS, pH 7.4; 20, 40, 80, and 100 mM imidazole), proteins were eluted with elution buffer (PBS, 250 mM imidazole, pH 7.4). Eluted proteins were dialyzed at 4 °C overnight against PBS (pH 7.4) in Snakeskin dialysis tubing (MWCO 10 kDa, Pierce). Eluted proteins were stored at −20 °C until use.

### Purification of Ni-NTA agarose-purified PICP using anti c-myc antibody-conjugated agarose

The Ni-NTA agarose-purified PICP (1 mg in 500 µL PBS) was added to 1 mL of anti-c-myc antibody-conjugated agarose bead (A7470, sigma) and incubated for 2 h at 4 °C under gentle rotation (4 r.p.m.). Then the sample was loaded to an empty chromatography column and flow-through was collected as the unbound fraction. Column was washed with 10 mL of washing buffer (PBS, pH 7.4) and then bound proteins were eluted with 5 mL of glycine buffer (0.1 M glycine, 0.1 M NaCl, pH 3.0). Eluted proteins (the bound fraction) were pooled. The unbound and bound fractions were dialyzed against at 4 °C overnight PBS (pH 7.4) and then concentrated to 1 mL using a Centricon concentrator with a 30-kDa molecular weight cut-off filter (Amicon). The bound and unbound fractions were then subjected to western blotting using anti-His and anti-c-myc antibodies.

### Western blotting of the purified proteins

The purified recombinant proteins (4 µg/lane) were subjected to SDS-PAGE using 10% gels and then transferred to polyvinylidene difluoride membranes. Non-specific binding was blocked for 1 h with 2% bovine serum albumin (BSA) in PBS (pH 7.4) containing 0.1% Tween 20 at 25 °C. The membranes were incubated overnight in a solution containing anti-His antibody (Santa Cruz Biotechnology, sc-803) or anti-c-myc antibody (Santa Cruz Biotechnology, sc-40). The bands were visualized with ECL western blot detection kit (GE Healthcare)^35^.

### SEC analysis

The purified PICP was analyzed in the Pharmacia AKTA FPLC system using a Superdex^TM^ 200 10/300 GL (10 × 300 mm; GE Healthcare) size exclusion column. After 1 mL injection of the purified PICP (2 mg/mL), the protein was eluted with TBS buffer (500 mM NaCl, 2.7 mM KCl, 12 mM Tris, pH 7.4) at a flow rate 0.35 mL/min. The purified PIIICP was analyzed in the Agilent 1160 HPLC system using the Superdex^TM^ 200 10/300 GL column (GE Healthcare). After 50 µL injection of the purified PICP (2 mg/mL), the protein was eluted with PBS buffer (pH 7.4) at a flow rate 0.75 mL/min. Chromatograms were obtained by monitoring the absorbance at 280 nm. To determine the apparent molecular mass of the protein, standard molecular weight markers (Blue dextran, 3000 kDa; alcohol dehydrogenase, 150 kDa; bovine serum albumin, 66 kDa; cytochrome c, 12 kDa; Sigma-Aldrich) were used.

### SDS-PAGE analysis

The purified proteins (each 1 µg/lane) were electrophoresed on 9% SDS-PAGE gels under reducing and non-reducing conditions. The gel was stained with InstantBlue protein stain (ISB1L, expedeon), according to the manufacturer’s instructions. The relative band intensity was measured by quantifying each band intensity on the gels using the ImageJ program (NIH).

### SDS-CGE analysis

Separation of the purified proteins was performed on an Agilent 2100 bioanalyzer (Agilent Technologies) in combination with the Protein 230 assay kit and the accompanying software, according to the manufacturer’s instructions. Briefly, samples were denatured by mixing in a 2:1 ratio with the sample buffer provided with the Agilent Protein 230 assay kit, and then heating in boiling water for 3 min. The denatured samples were then diluted 1:15 in deionized water and loaded onto the primed chip. The protein size marker and the internal markers were used as a reference for sizing and relative quantification. The bioanalyzer software automatically calculated the size and concentration of each separated peak. The amount of each protein in the sample expressed as percentage of total protein could be determined directly from the software.

### Selection of anti-PICP scFv from phage library

A phage library representing over 7.6 × 10^9^ independent human scFvs was provided by Prof. Hyun-bo Shim (Ewha Womans University, Korea)^16^. The phage library was screened against recombinant PICP using recombinant PIIICP as a soluble competitor following procedures described previously^16^. After four rounds of panning, phagemid vectors were extracted from high affinity binding phages and then subjected to DNA sequencing. Expression and purification of isolated scFv clones were also performed as described previously^16^.

### Expression and purification of humanized IgG1 antibody

The isolated scFv clones (2D and 4 G) were reformatted into IgG1 by sub-cloning the respective VH and VL genes into the modified pcDNA 3.4 vector (Invitrogen) carrying the human IgG1 Fc (CH1-hinge-CH2-CH3) gene and CL gene, respectively^17^. Antibodies were produced by transient transfection of the plasmids into HEK293F cells using the Freestyle 293-F expression and media system (Invitrogen), as described in previous studies^17,35^. Antibodies were purified from the culture supernatants using a CaptivA™ PriMAB protein A column (Repligen) following the manufacturer’s protocol, and then stored in PBS (pH 7.4) at −20 °C until further use. The concentration of mAbs was determined with the BCA protein assay kit (Pierce, 23225).

### Rabbit immunization and polyclonal antibody preparation

The polyclonal PICP antibody was raised by immunizing rabbits in Young-In Frontier Co. (Seoul, Korea). The immunization was repeated at days 21, 35, 49, and 63 after the first injection with Freund’s incomplete adjuvant. The animals were bled one week after the fifth injection and the sera were collected by centrifuging the clotted blood at 2,000 *g*. Following IgG purification using the protein A column, the anti-PICP pAb was further purified by PICP affinity chromatography. The pAb in PBS (pH 7.4) was stored at −20 °C until use. The concentration of pAb was determined with the BCA protein assay kit (Pierce, 23225).

### Binding analysis by ELISA

96-well microtiter plates were coated with 100 μL of human recombinant PICP (4 µg/mL) at 25 °C in PBS (pH 7.4) for 1 h, and then blocked with blocking buffer (1% BSA diluted in PBS, pH 7.4) for 1 h at 25 °C. After washing 3 times with PBST (PBS, pH7.4, 0.05% Tween 20), serially diluted scFv or IgG clones in blocking buffer were added and incubated for 1 h at 25 °C. The wells were then washed 3 times with PBST. Bound scFvs or mAbs were detected with 100 μL of HRP-conjugated anti-HA antibody (Roche Applied Science) or HRP-conjugated anti-human IgG antibody (GeneScript). Subsequently, enzymatic reactions were carried out at 25 °C by adding TMB (3,3′,5,5′-tetramethylbenzidine) for 5–15 min, and stopped by adding 1 N H_2_SO_4_. Absorbance was read in a 96-well plate reader at 450 nm subtracting the background measurement at 620 nm.

### SPR analysis

Kinetic interactions of 2D and 4 G mAbs with recombinant PICP were measured at 25 °C using BIAcore 2000 SPR biosensor (GE Healthcare), as described previously^17,35^. Briefly, after immobilization of the recombinant PICP onto the carboxymethylated dextran surface of a CM5 sensor chip at a level of about 185 response units (RUs), antibodies (100 nM to 6.25 nM) were injected into the flow cell for 3 min at a flow rate of 30 μL/min, with a 3-min dissociation period per cycle. After each cycle, the surfaces were regenerated with a buffer (20 mM NaOH, 1 M NaCl, pH 10.0). The values of the dissociation (*k*_d_) and association rate constants (*k*_a_), and the dissociation equilibrium constant (*K*_D_) were determined using the BIAevaluation software provided by the instrument manufacturer.

### Immunoprecipitation of PICP

Immunoprecipitation (IP) was performed to analyze whether the 2D mAb can bind to the native PICP. Human normal dermal fibroblast cells (ATCC PCS-201-012™) cultured in Dulbecco’s modified Eagle’s medium (DMEM) supplemented with 10% fetal bovine serum, 1% penicillin/streptomycin, and 1 mL of fibroblast culture supernatant (serum free) were incubated with 4 μg of 2D mAb or IgG1 isotype control for 16 h at 4 °C, and then subjected to immunoprecipitation for 2 h at 4 °C with protein A agarose (50% slurry, amicogen, 5010005) to pull down the antibodies (2D mAb and IgG1 control). The immune complexes were subsequently washed with 1× RIPA lysis buffer (20 mM Tris-HCl pH 7.5, 150 mM NaCl, 1 mM EDTA, 1 mM EGTA, 1% NP-40, 1% sodium deoxycholate, and protease inhibitor; Thermo Scientific, 78440), and equal precipitates were analyzed by western blotting with the anti-PICP rabbit pAb that was raised against recombinant human PICP in this study.

### LC/MS analysis

The gel band (from IP result) was eventually digested by the in-gel trypsin digestion process, following previous procedures^36^. Peptide samples were analyzed on an LTQ-Orbitrap Velos (Thermo Fisher Scientific) connected to an Easy-nano LC II system (Thermo Fisher Scientific) incorporated with an autosampler. The dried peptide samples were resuspended in 70 μL of 0.1% formic acid, and an aliquot (7 μL) was injected into a reversed-phase peptide trap EASY-Column (L 2 cm, ID 100 μm, 5 μm, 120 Å, ReproSil-Pur C18-AQ, Thermo Fisher Scientific) and a reversed-phase analytical EASY-Column (L 10 cm, ID 75 μm, 3 μm, 120 Å, ReproSil-Pur C18-AQ, Thermo Fisher Scientific), and electrospray ionization was subsequently performed using a 30 μm (i.d.) nano-bore stainless steel online emitter (Thermo Fisher Scientific). The total duration of LC gradient analysis was 60 min. The peptides were eluted in a linear gradient of 10–40% buffer B over a period of 40 min, with buffer A (0.1% formic acid in H_2_O) and buffer B (0.1% formic acid in acetonitrile), and a flow rate of 0.3 μL/min. The temperature and voltage applied to the capillary was 275 °C and 1.9 V, respectively. All data were acquired with the mass spectrometer operating in automatic data-dependent switching mode. The MS survey was scanned from 350 to 2000 m/z with the resolution set to 100,000.

### MS/MS data analysis

All MS/MS samples were analyzed with Sequest (XCorr Only) (version v.27, rev. 11) and X! Tandem [version CYCLONE (2010.12.01.1)] using the UniProt human database (version 2014). Search parameters were set as described below. The cleavage site was fully digested using trypsin/Lys-C (after KR/−) with up to 2 missed cleavage. The precursor and fragment mass tolerance were 25 ppm and 1.0 Da, respectively. Carbamidomethylation (+57.021 Da) of cysteine (C) was set in Sequest (XCorr Only) and X! Tandem as a fixed modification. Oxidation (+15.995 Da) of methionine (M) was set in Sequest (XCorr Only) and X! Tandem as a variable modification. Scaffold searched the additional variable modifications: Glu → pyro-Glu (−18.01), ammonia-loss (−17.03), and Gln → pyro-Glu (−17.03). Each processed data were subsequently transformed to an *.sf file with Scaffold 4 Q + S program (version 4.6.1, Proteome Software Inc.). Scaffold program was used to validate MS/MS-based peptide and protein identifications, and to process the quantitative analysis. Peptide identifications were accepted if they could be established at greater than 90.0% probability by the Peptide Prophet algorithm^37^ with Scaffold delta-mass correction. Protein identifications were accepted if they could be established at greater than 90.0% probability and contained at least 2 identified peptides. Protein probabilities were assigned by the Protein Prophet algorithm^38^. Proteins that contained similar peptides and could not be differentiated based on MS/MS analysis alone were grouped to satisfy the principles of parsimony.

### Preparation of LX-2 cellular extracts and culture supernatants for PICP measurement

The human hepatic stellate LX-2 cell line was kindly provided by Prof. Sang Geon Kim (Seoul National University, Korea). LX-2 cells were cultured in DMEM containing 10% fetal bovine serum and 1% penicillin/streptomycin at 37 °C in a 5% CO_2_ incubator^23^. To induce collagen expression, LX-2 cells (8 × 10^6^ cells) seeded in 100 mm dish were cultured for 12 h, and then starved for 12 h in serum-free DMEM. The cells were then cultured in serum-free DMEM supplemented with 2, 5, 10 ng/mL recombinant human TGF-β (R&D systems, 240-B) for 12 h. The cell culture supernatants were prepared by centrifugation, filtered (Corning, 431219) and subjected to sandwich ELISA, as described below. Cells were washed with ice-cold PBS (pH 7.4) and lysed in lysis buffer [0.05% SDS, 0.5% Triton X-100, 10% glycerol, 50 mM Tris-HCl (pH 7.4), 100 mM NaCl, 1 mM EDTA, and protease inhibitor; Thermo Scientific, 78440]. The detergent-insoluble materials were removed by centrifugation at 12,000 *g* for 10 min at 4 °C. The soluble cellular extracts were subjected to analysis by western blotting and sandwich ELISA, as described below. Protein concentrations were measured using the BCA protein assay kit (Pierce, 23225). Western blotting was performed using specific antibodies and proteins, and visualized using ECL^TM^ western blotting detection reagents (GE Healthcare, RPN2209)^35^. Equal amounts of lysates were analyzed by western blotting with β-actin as a loading control. The antibodies used in this study were as follows: COL1A1 (DSHB, SP1.D8) for type 1 procollagen, p-Smad2 (Cell Signaling Technology, 3108), and β-actin (Santa Cruz Biotechnology, sc-47778). For quantification of western blotting data, band intensities were quantified using the Gel-Pro Analyzer software and normalized to the loading control^35^.

### Sandwich ELISA assay

96-well microtiter plates were coated with 100 µL of 2D mAb (4 μg/mL) and incubated overnight at 4 °C. The wells were blocked with blocking buffer (1% BSA diluted in PBS, pH 7.4) for 1 h at 25 °C and washed 3 times with PBST. Serially diluted samples (final 20 µL) of recombinant human PICP, LX-2 fibroblast samples (20 µL of the cell extracts or the culture supernatants), or a human serum sample (S1-100ML, Millipore) (1, 2, 5, or 10 µL of serum diluted in PBS to be total 20 µL) were added and incubated for 1 h at 37 °C. Plates were washed 3 times with 200 µL of PBST, and 100 μL of anti-PICP rabbit pAb in PBST was added and incubated for 1 h at 25 °C. The plates were then washed 3 times with 200 µL of PBST, followed by incubation with HRP-conjugated anti-rabbit antibody (Millipore, AP187P) at 25 °C for 1 h. Subsequently, enzymatic reactions were carried out at 25 °C by incubation with TMB for 5-15 min, and stopped by adding 1 N H_2_SO_4_. Absorbance was read in a 96-well plate reader at 450 nm subtracting the background measurement at 620 nm. The standard curve was fitted by a 4PL model^19^. The LoD, which is the lowest concentration of analyte at which the response can be reliably distinguished from the background noise, was calculated by the standard formula: [LoD = average absorbance of the blank +3 × (SD_blank_) (10 replicates)]^19,21^. The recovery rate was calculated as a percentage by the formula: [the obtained value/expected value × 100]^21^, where the expected value of sample was determined using a BCA Kit (Pierce, 23225). The CV value was determined by the equation: [SD of the mean/the mean value × 100]^19,21^. The commercially available Takara sandwich ELISA kit (Cat. #MK101) was purchased from Takara Korea Biomedical Inc., and the assay was performed according to the manufacturer’s instructions.

To validate the repeatability of the developed ELISA, the intra- and inter-assay CV values were determined by measuring the concentrations of recombinant human PICP spiked in PBS buffer at three different doses^18,21^. Intra-assay variability was calculated from the four repeated measurements of three different doses of samples on a single day but at different time points, while inter-assay variability was calculated in three independent experiments (n = 3~4).

### Statistical analysis

Data represent the mean ± SD of at least 3 independent experiments that were performed in triplicate, unless otherwise specified. Data from test and control groups were compared and analyzed for statistical significance by a two-tailed, unpaired Student’s *t*-test using MS Excel.

### Data availability

All data in this study are available within the article or from the authors on request.

## Electronic supplementary material

Supplementary information
