# Supplementary material for: Production of recombinant human procollagen type I C-terminal propeptide and establishment of a sandwich ELISA for quantification
Source: Sci Rep. 2017 Nov 21;7:15946. doi: 10.1038/s41598-017-16290-9 (PMC5698462; doi:10.1038/s41598-017-16290-9)
Supplement: Supplementary file 1 — Supplementary information [file 41598_2017_16290_MOESM1_ESM.pdf]

Production of recombinant human procollagen type I C-terminal propeptide and establishment of a sandwich ELISA for quantification

Woo-Young Seo[1,§], Jeong-Ho Kim[2,§], Du-San Baek[2], Su-Jung Kim[2], Su-Jin Kang[1], Won Suk Yang[1], Ji-Ae Song[1], Moo-Seung Lee[3], Sunghoon Kim[1,*] and Yong-Sung Kim[2,*]

[1]Medicinal Bioconvergence Research Center, Seoul National University, Suwon 16229, Korea.
[2]Department of Molecular Science and Technology, Ajou University, Suwon 16499, Korea.
[3]Infectious Disease Research Center, Korea Research Institute of Bioscience and Biotechnology,
Daejeon 34141, Korea.

Inventory of Supplementary Information
Supplementary Figure 1
Supplementary Figure 2
Supplementary Figure 3
Supplementary Figure 4
Supplementary Figure 5
Supplementary Figure 6
Supplementary Figure 7

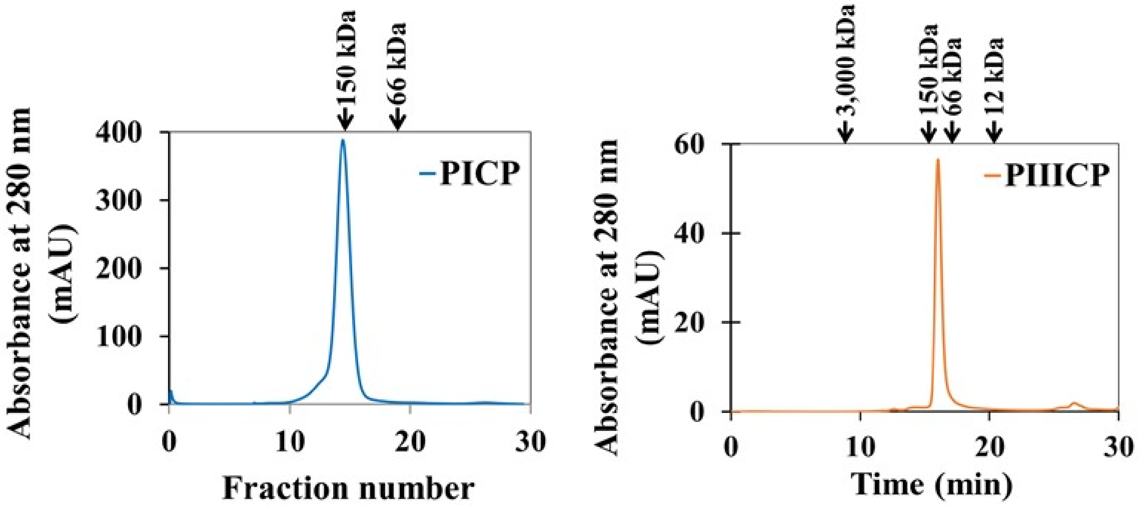

Supplementary Figure 1. SEC elution profiles of the purified recombinant PICP (a) and PIIICP (b). The purified PICP (1 mg injection) and PIIICP (0.1 mg injection) was injected into an FPLC system and an HPLC system, respectively, which equipped with a Superdex^{TM} 200 10/300 GL (10 mm × 300 mm; GE Healthcare) size exclusion column. The relatively high amount of purified PICP (1 mg) was injected to monitor the presence of unassembled free dimers or monomers. Arrows indicate the eluted positions of the molecular weight standards (blue dextran, 3,000 kDa; alcohol dehydrogenase, 150 kDa; bovine serum albumin, 66 kDa; cytochrome c, 12 kDa).

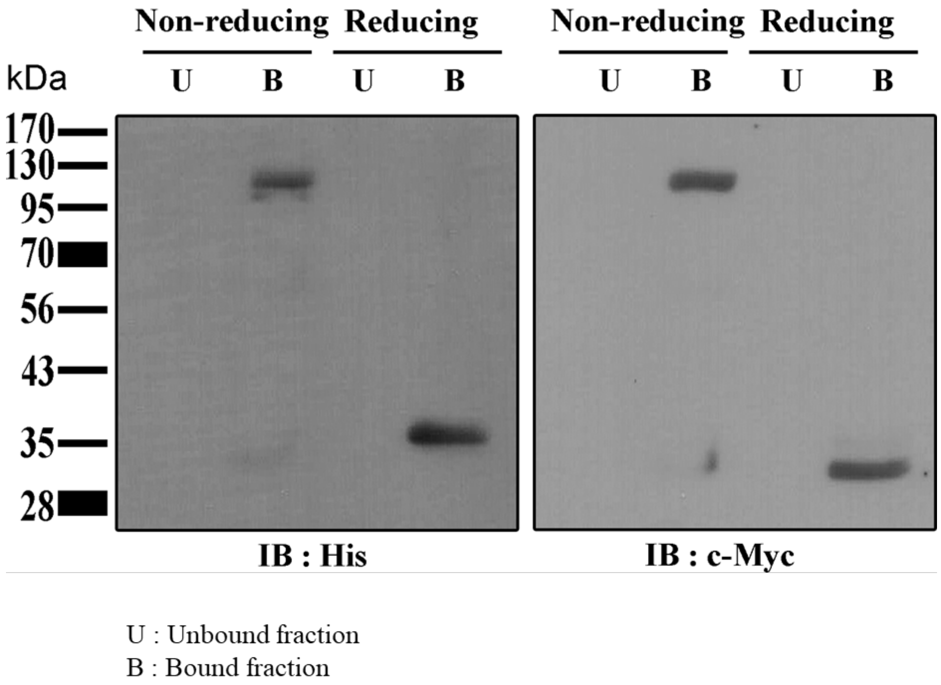

Supplementary Figure 2. Western blot analysis of one step further purified PICP.
The purified PICP using Ni-NTA agarose column was further purified with an anti-c-myc antibody-conjugated agarose bead to monitor free monomers or homotrimers. The bound and unbound fractions were subjected to SDS-PAGE in non-reducing or reducing conditions, and the gel was blotted with anti-His antibody (left) and anti-c-myc antibody (right).

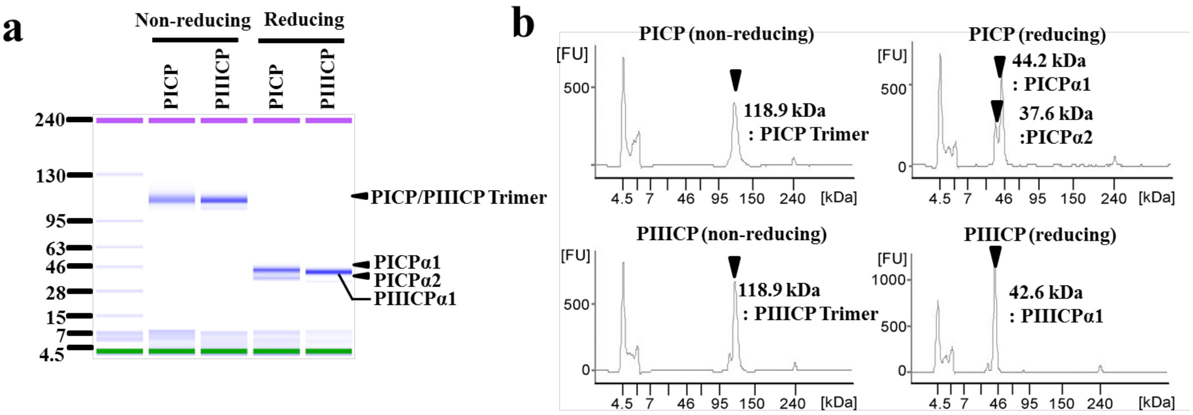

Protein	condition	Total Rel. Conc. (ng/μl)	Rel. Conc. (ng/μl)
PICP	Non-Reducing	1,351 (118.9 kDa)	1,331 (98.5%)
	Reducing	2,055 (43.6;37.4 kDa)	1,414 (68.8%) 618 (30.1%)
PIIICP	Non-reducing	1,626 (118.9 kDa)	1,424 (87.6%)
	Reducing	2,000 (42.1 kDa)	1,856 (92.8%)

Supplementary Figure 3. Another example of SDS-CGE analysis of purified recombinant PICP and PIIICP, shown in Fig. 2b,c. (**a**) Gel-like image of SDS-CGE analysis of the purified recombinant PICP and PIIICP. (**b**) Electropherogram of SDS-CGE analysis of the recombinant PICP and PIIICP shown in (**a**). Significant peaks are marked with black arrow heads, with the calculated molecular weights. The inset table below shows the precise quantity and ratio of each peak area in the electropherogram.

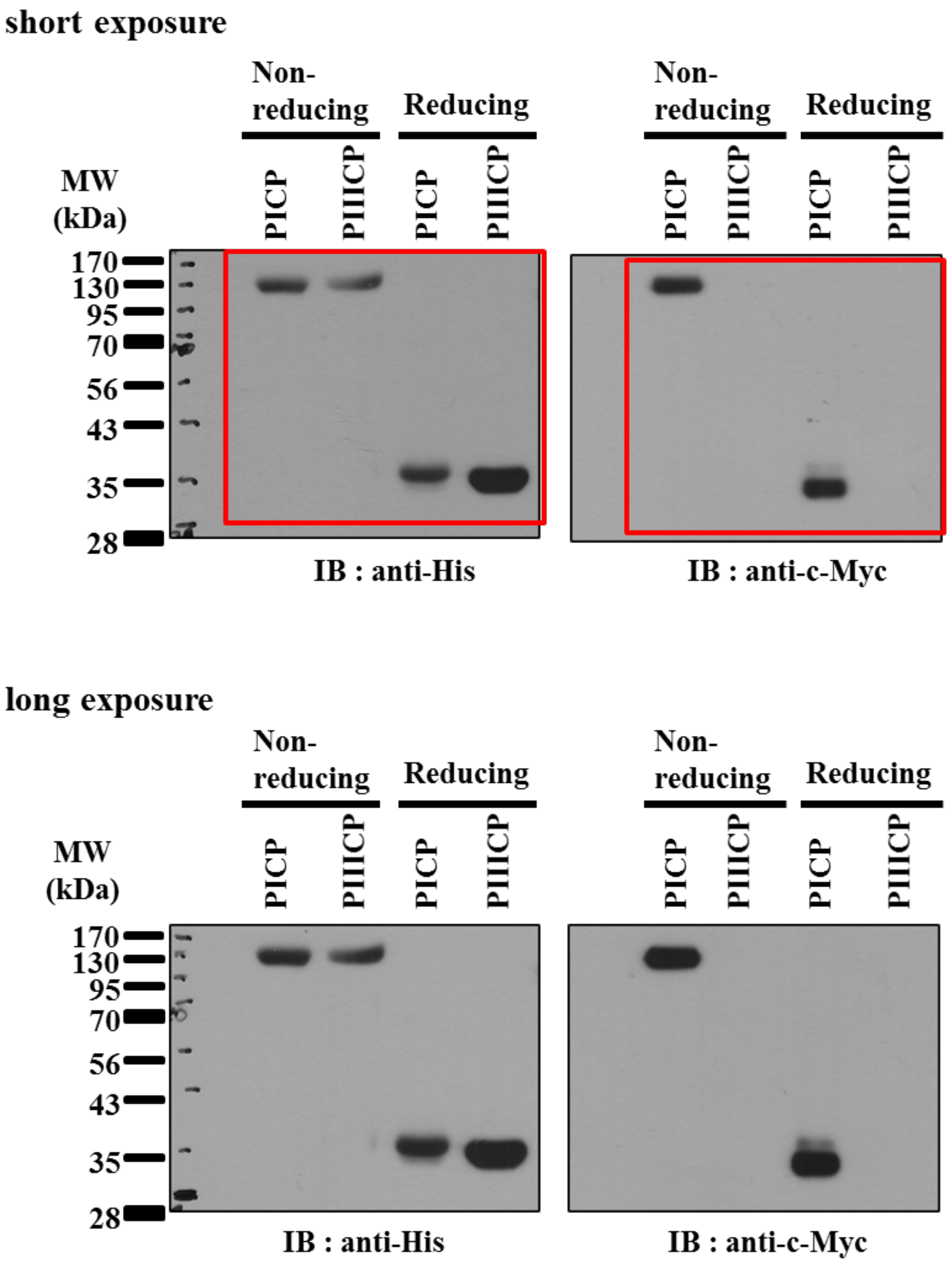

Supplementary Figure 4.

Full-length blots corresponding to Fig.1d. Indicated parts (red box) are shown in Fig.1d.

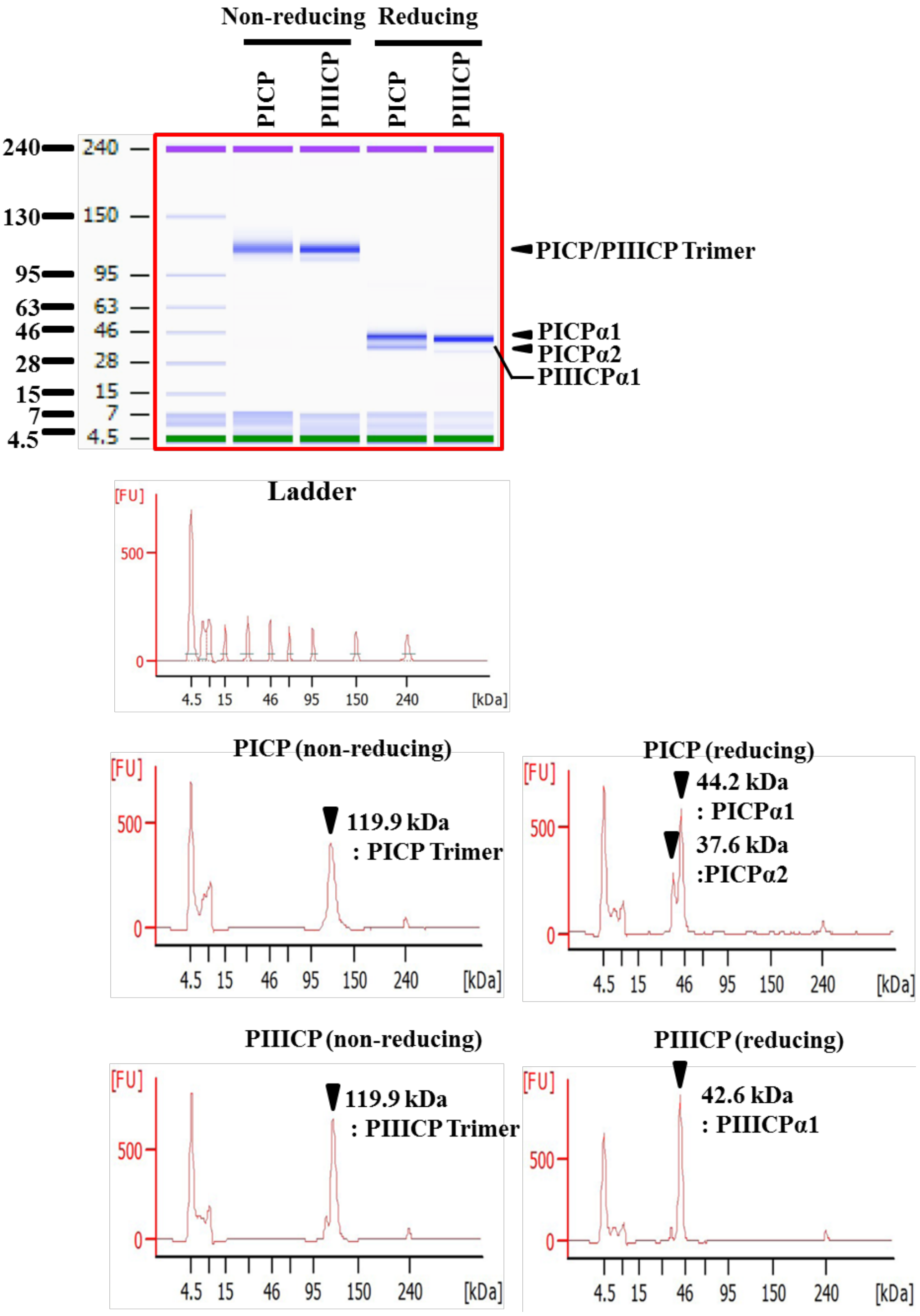

Supplementary Figure 5.
Indicated parts (red box and red graph) are shown in Fig.2b, c.

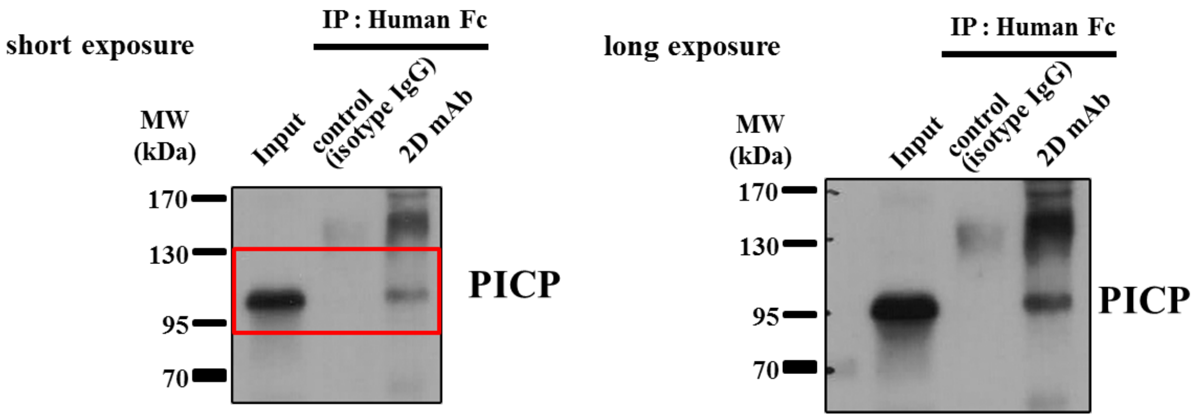

Supplementary Figure 6.

Full-length blots corresponding to Fig.4a. Indicated parts (red box) are shown in Fig.4a.

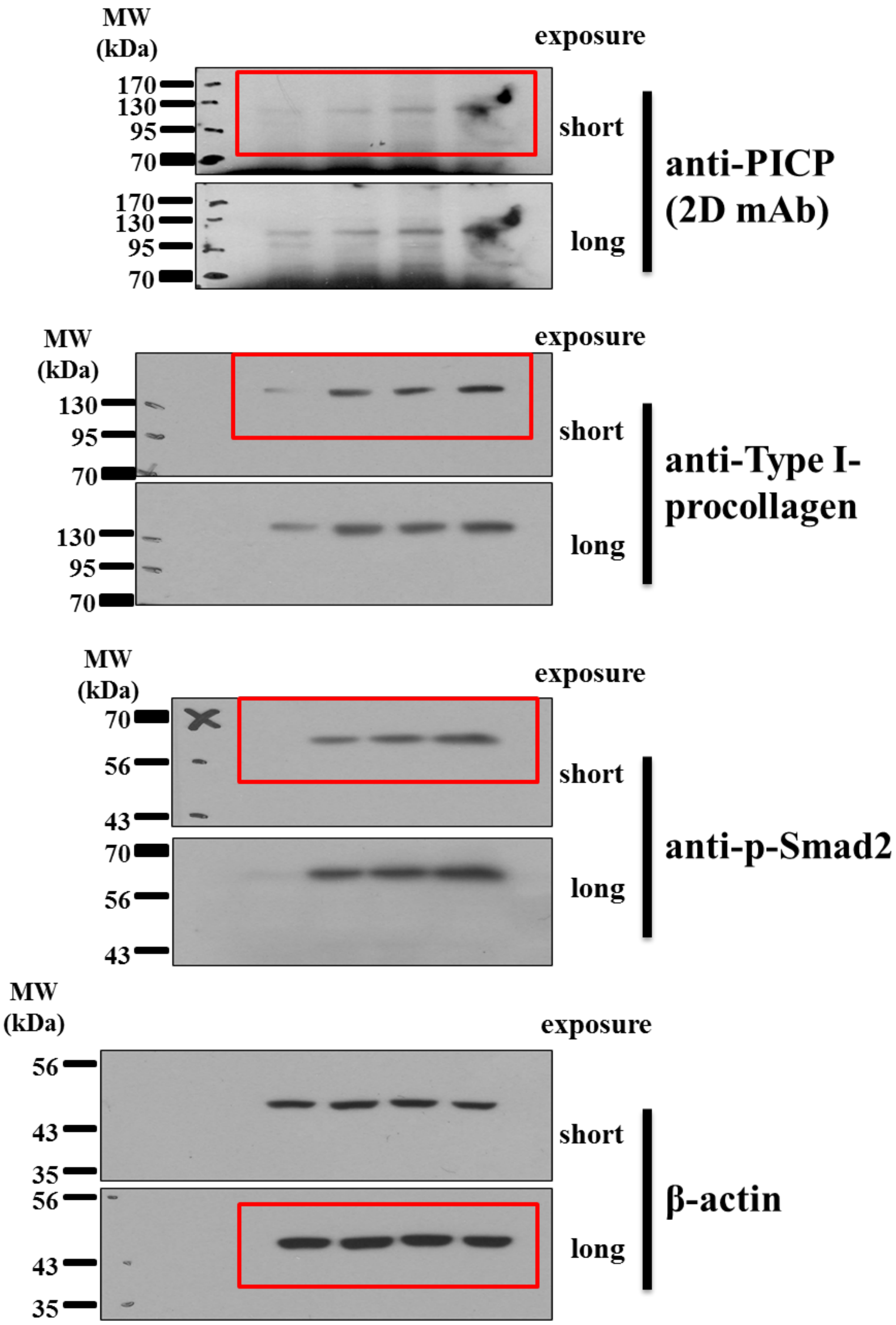

Supplementary Figure 7.

Full-length blots corresponding to Fig.5c. Indicated parts (red box) are shown in Fig.5c.
